# Samaritans Ancient and Modern

**Published:** 1892-06-18

**Authors:** 


### The Hospital

AN INSTITUTIONAL JOURNAL OF

**Science, Medicine, Nursing, and Philanthropy.**

*For Hospitals, Asylums, Infirmaries, Dispensaries, Medical Practitioners, Students, Nurses, and the Charitable Public.*

Vol. XII.—No. 299.] [June 18, 1892.

### SAMARITANS ANCIENT AND MODERN.

If a man have a fractured skull and broken ribs that are driving into his lungs every time he takes a breath, it matters little to him whether he came by his injuries at the hands of thieves as he journeyed from Jerusalem to Jericho, or in a fall from a scaffold four stories high as he plied his trade of bricklayer on a house in modern London. Nor is there any difference, so far as we can see, between the act of the sympathetic Samaritan who picked up a wounded traveller, soothed him, and took him to the nearest place of refuge and that of the kindly hospital, with its comfortable bed, tender nurse, and skilful surgeon, which receives the London bricklayer after his headlong fall upon the pavement. And yet the story of the former has proved such a moving tale, that painters have painted it, and men and women of all ranks have looked upon the picture with melting eyes for nineteen completed centuries. It is not given to every man to tell an immortal story even when he has the materials at his fingers' ends. If it were, one might surely hope to strike a sympathetic chord in the heart of every reader by relating the simple fact that the London hospitals have enacted the part of the Good Samaritan seventy-nine thousand two hundred times during the past year.

When a man quotes the proverb, "Actions speak louder than words," everybody instantaneously agrees with him. But it has happened ten thousand times in the history of the world that great actions have had no power to move at all, and that the word of genius falling upon the ear has kindled every eye and fired every heart. The seventy-nine thousand in-patients received at the London hospitals during the past year had every one a separate tale to tell of pain and trouble. Those seventy-nine thousand were not out-patients who could walk into the hospital, tell their story, receive their medicine, and go their way, often to be heard of no more. They were in-patients, men, women, and children suffering from prolonged and serious illness; many of them as badly injured as the traveller between Jerusalem and Jericho, and some of them far more fatally ill. How many gracious women have watched through the lonely hours of the night by the side of the suffering child or the anguished and dying man! How many strong-hearted surgeons, grey in experience, have fought grim fights with death and conquered when hope seemed gone! And yet there is no evidence to show that the five millions of London have even been so much as conscious of the idylls that were being worked out in their midst, or have paid one extra moment's attention to these strange and moving actions that are believed to spea much louder than words!

At one hospital alone 682 cases of cancer were treated last year. Many of these were placed under anæsthetics, and thus brought immediately to the door of death. They had to undergo maiming operations, to be followed by weeks of slow and feeble convalescence, and to be dismissed with the feeblest of all hopes of ultimate recovery. Most of these poor creatures were women. The story of many was tragical; not tragical to themselves only, but to the doctor who had to treat them with little hope, and to the nurse who had to nurse them, knowing full well that the nursing was often but a gradual preparation for the final doom. Yet nurse and doctor both did their duty cheerfully, soothingly, with kind words and gentle ministrations, thereby smoothing the rough and thorny pathway to premature death. Those 682 represented but a portion of the annual victims of cancer that are treated in London alone. Every hospital of any magnitude has its surgical wards, in each of which more or fewer cancer cases may almost always be found.

There are several chest hospitals in London. In one of these, the Hospital for Consumption, nearly 15,000 patients were treated last year, of whom 1,594 were so seriously ill as to require to be taken into the hospital for a long period. The present writer has always considered that a physician who devotes himself entirely to consumptives must have a life almost too sad for mortal man. It is inevitable that the life of every doctor and nurse of human feeling must be dashed with many a ray of sadness. But to live with consumptives and for consumptives always involves a depth of melancholy which those who are ignorant of hospitals and diseases, can form no conception of. As in the case of the one cancer hospital singled out, so this one hospital for consumption receives but a portion of the consumptives of London and its vicinity. The whole number of victims is counted by thousands annually. Many consumptives happily are cured. But even in the cases of those who are not, what a satisfaction it is to know that their sufferings are alleviated by every resource of the most perfect skill of the times.

In one women's hospital last year 942 babies first saw the light. Next to dying, in thousands of the poorest homes in London, the most melancholy circumstance is that of giving birth to a child. Even the animals in the open field are happier than human creatures who have to bring forth their children in one room, surrounded immediately by their families, and all but immediately by the old and young of both sexes in scores and sometimes in hundreds. No hospitals in London make less of their work than those that provide for women, but to none is a refuge in distress and trouble more welcome than to the hard-pressed and brain-harassed mother of a family. The Lying-in Hospitals are among the most entirely indispensable of London's noble charities.

Is there a father or a mother anywhere who will not be touched by the knowledge that one single children's hospital took into its wards for prolonged treatment no fewer than 1,495 children last year? On this text a volume might be written. The sick and suffering child in a single-roomed house is a prey to every conceivable evil. To remove him from filth, poverty, and neglect to the clean comfort of the hospital bed and ward is almost like opening the gate of heaven to him. For him the hospital ward is better than the Queen's Palace.

As the result of a detailed enquiry made at the close of last year, we announced the striking fact that not less than a quarter of a million accident cases are brought to the London hospitals annually. One General Hospital alone treated more than 20,000, many of them of the most severe and dangerous character. Of the out-patients we hardly dare venture to speak, they are so numerous. The number of out-patients treated last year at the various hospitals reached the enormous total of one million one hundred and eighty thousand eight hundred and forty-five; as many as a third of the whole population of Scotland. We have picked out here and there a mere half dozen facts which serve as samples of the work which is constantly being done in London for the sick poor. Detailed and tabulated statements representing work, of which the cases we have selected are but as a few lines of the index to many volumes, will be found on page 189 of the present issue.

What do all these figures, amounting almost to millions, mean—in medical skill, gentle nursing, sheltering the homeless, repairing waste, and restoring health to so many of Society's hardly-used conscripts? They mean a work of benevolence and skill which has never had a parallel in the history of the world. They mean the silencing of such a mighty groan of human anguish as might rend the very heavens by its unspeakable intensity. They turn what would otherwise be one of the darkest and most saddening blots on our civilization into one of its most pleasing and satisfying pictures. They give as much contentment to the consciences of the rich as they give help and healing to the poor. The churchyard used to be called "God's acre"; the hospitals may be called "God's harbour" for those who have been wrecked and all but lost on the stormy sea of life. These harbours of refuge must be maintained, and kept open and free for all comers. It is true that a deficiency of £147,000 stares us in the face as the result of last year's working, and some timid people are overwhelmed by the magnitude of this sum. But if once the good will of the whole of London would rouse itself from its slumber, such a paltry deficit would be swept away like a feather on a swift mountain torrent. All lovers of hospitals must stand together and press on the great work of instructing and enlightening the public. Nowhere can this be more effectually done than by competent addresses from the pulpit. The present hour seems dark to some. But dark or bright, the hospitals are the necessity of the poor and the glory of the rich, and however strenuous be the effort needed for their increasing maintenance, it must never be relaxed until they are landed high and dry on the shores of a permanent prosperity.

